# Transcriptomic and Targeted Metabolomics Analysis of Detached *Lycium ruthenicum* Leaves Reveals Mechanisms of Anthocyanin Biosynthesis Induction through Light Quality and Sucrose Treatments

**DOI:** 10.3390/metabo13091004

**Published:** 2023-09-11

**Authors:** Haitao Zeng, Tao Zheng, Xue Peng, Qi Tang, Hao Xu, Mengjiao Chen

**Affiliations:** 1 School of Biological Science and Engineering, Shaanxi University of Technology, Hanzhong 723001, China; zenghaitao@snut.edu.cn (H.Z.); 18329616748@163.com (X.P.); tangqi@snut.edu.cn (Q.T.); xh2003@126.com (H.X.); cmj18168212780@163.com (M.C.); 2 Shaanxi Province Key Laboratory of Bio-Resources, Hanzhong 723001, China; 3 Qinba Mountain Area Collaborative Innovation Center of Bioresources Comprehensive Development, Hanzhong 723001, China; 4 Qinba State Key Laboratory of Biological Resources and Ecological Environment (Incubation), Hanzhong 723001, China

**Keywords:** *Lycium ruthenicum*, anthocyanins, transcriptomic analysis, light quality, sucrose osmotic stress

## Abstract

Light quality and sucrose-induced osmotic stress are known to cause anthocyanin synthesis in detached *Lycium ruthenicum* leaves. To identify the mechanisms by which the kind of light quality and sucrose concentration are induced, here, we conducted transcriptome sequencing in detached *L. ruthenicum* leaves treated with different qualities of light and sucrose concentrations. Leaves treated with blue light or sucrose showed a significantly increased total anthocyanins content compared to those treated with white light. Delphinidin-3-O-rutinoside and delphinidin-3-O-glucoside production were differentially regulated by the BL(−S), BL(+S), and WL(+S) treatments. The structural genes *CHS*, *CHI*, *F3′H*, *F3′5′H*, *ANS,* and *UFGT* were significantly up-regulated in leaves treated with blue light or sucrose. Leaves treated with blue light additionally showed up-regulation of the light photoreceptors *CRY1*, *PIF3*, *COP1*, and *HY5*. The anthocyanin-related genes *NCED1*, *PYR/PYL*, *PP2C*, *SnRK2*, and *ABI5* were significantly up-regulated in leaves treated with sucrose, promoting adaptability to sucrose osmotic stress. Co-expression and *cis*-regulatory analyses suggested that *HY5* and *ABI5* could regulate *LrMYB44* and *LrMYB48* through binding to the G-box element and ABRE element, respectively, inducing anthocyanin synthesis in response to blue light or sucrose treatment. Candidate genes responsive to blue light or sucrose osmotic stress in the anthocyanin biosynthesis pathway were validated through quantitative reverse transcription PCR. These findings deepen our understanding of the mechanisms by which blue light and sucrose-induced osmotic stress regulate anthocyanin synthesis, providing valuable target genes for the future improvement in anthocyanin production in *L. ruthenicum*.

## 1. Introduction

*Lycium ruthenicum Murray*, a multi-spiny shrub in the family *Solanaceae*, is widely distributed in the desert regions of northwest China. It is a pioneer species, an indicator plant of saline-alkali soils, and has strong a windbreak and sand fixation ability, in addition to salt and drought resistance [1]. Consumers enjoy *L. ruthenicum* due to its abundance of compounds that confer anti-oxidation, anti-inflammatory, anti-aging, and anti-diabetes properties, including anthocyanins, polysaccharides, amino acids, phenols, and flavonoids [2,3]. A total of 27 anthocyanins have been identified in *L. ruthenicum* to date, which together give *L. ruthenicum* berries the highest total anthocyanin content of all known berries. Therefore, the anthocyanin content is a key indicator to reflect the functional value of *L. ruthenicum*.

The mechanisms of anthocyanin synthesis and regulation have been comprehensively delineated in many plants. Specifically, anthocyanins are known to be synthesized via the flavonoid pathway and to be generated through the activities of a series of structural genes [4,5], including chalcone synthase (*CHS*), chalcone isomerase (*CHI*), flavanone 3-hydroxylase (*F3H*), dihydroflavonol 4-reductas (*DFR*), anthocyanin synthetase (*ANS*), and UDP-glucose: flavonoid 3-O-glucosyl transferase (*UFGT*). Anthocyanin synthesis structural gene expression is coordinately regulated by the MBW transcriptional complex, which is composed of three types of proteins: R2R3-MYB transcription factors, basic helix-loop-helix (bHLH) transcription factors, and WD-repeat proteins [6]. The spatial and temporal expression patterns of anthocyanin synthesis structural genes are primarily determined by the activity of the R2R3-MYB transcription factors in this complex, with some individual gene family members regulating separate patterns [7]. Previous studies have indicated that anthocyanin can be triggered in *L. Ruthenicum*-detached leaves by specific light qualities or sucrose-induced osmotic stress. However, the specific light qualities and concentrations of sucrose that can induce such gene expressions remain unclear, as are the identities of the associated transcription factors.

Higher plants have evolved several types of photoreceptors for light sensing, which directly or indirectly activate the related structural genes and transcription factors in the anthocyanin synthesis pathway and promote anthocyanin accumulation [8]. Known photoreceptors include cryptochromes, which sense blue light (BL); phytochromes, which sense red light (RL) and far-red light (FRL); and UV RESISTANCE LOCUS 8 (UVR8), which senses ultraviolet (UV)-B light [9,10]. Anthocyanin biosynthesis appears to be most strongly induced by BL [11]. In red pear, BL enhances anthocyanin synthesis through the activity of the CRYPTOCHROME (CRY)-CONSTITUTIVELY PHOTOMORPHOGENIC 1 (COP1)-ELONGATED HYPOCOTYL 5 (HY5) module [12]. In eggplant (*Solanum melongena* L.), BL-triggered interactions between CRY1 and CRY2-COP1 promote binding of the transcription factors HY5 and MYB1 to downstream anthocyanin structural genes, inducing anthocyanin accumulation [13]. In tomato (*Solanum lycopersicum*), increasing the proportion of BL under which seedlings are grown increases *CRY1a* expression and anthocyanin contents [14]. Despite these known mechanisms in some plant species, a specific mechanism of BL-associated anthocyanin synthesis has yet to be reported in *L. ruthenicum*.

In recent years, many studies have shown that sucrose is an important signal involved in the regulation of various plant developmental processes, including anthocyanin biosynthesis. For example, experiments have shown that osmotic stress induced through treatment with glucose, sucrose, or mannitol can contribute to anthocyanin accumulation, with sucrose stimulating anthocyanin coloration to the greatest degree [15]. In a *Camptotheca acuminata* cell culture, treatment with high concentrations of sucrose increases anthocyanin content [16]. Solfanelli et al. (2006) [17] reported that flavonoid biosynthesis genes are significantly up-regulated in *Arabidopsis thaliana* in response to sucrose-induced osmotic stress, and proposed that interactions between sucrose and plant hormone signaling pathways regulate anthocyanin accumulation. Exogenous sucrose treatment significantly up-regulates anthocyanin synthesis genes and increases anthocyanin content in radish hypocotyls [18]. However, the regulation mechanism of anthocyanin synthesis in *L. ruthenicum* under blue light and sucrose osmotic stress was still unclear. Therefore, it was of great significance to explore the molecular mechanism of anthocyanin synthesis in detached *L. Ruthenicum* leaves with different light qualities and sucrose osmotic stress treatments.

In the present study, we identified key genes involved in light- and sucrose-responsive anthocyanin biosynthesis in the economically important species *L. ruthenicum*. Using detached leaves, we explored the effects of several light qualities and sucrose concentrations on anthocyanin accumulation. RNA-sequencing (RNA-seq) was conducted in parallel to characterize putative regulatory genes associated with anthocyanin accumulation in detached *L. ruthenicum* leaves in response to light and sucrose treatments. The results of this study laid a molecular foundation for further understanding the effect and regulation mechanism of blue light and sucrose-simulated osmotic stress on anthocyanin synthesis in detached *L. ruthenicum*. 

## 2. Materials and Methods

### 2.1. Experimental Materials

*L. ruthenicum* seeds were obtained from the Ningxia Academy of Agriculture and Forestry Sciences and sterilized using high-pressure sterilizer. Murashige and Skoog (MS) medium was prepared by combining 4.74 g of MS powder, 20.00 g of sucrose, 7.00 g of agarose powder, and deionized water to 1 L. The medium was autoclaved at 121 °C for 15 min and then poured into plates. On an ultra-clean bench, sterilized seeds were evenly distributed on the solid MS medium with *L. ruthenicum* seeds per plate. The plates were incubated at 25 °C for 60 d under a 16/8 h light/dark photoperiod. The leaves of the sterile seedlings were then used in subsequent experiments as described below.

### 2.2. Light Quality and Sucrose Treatments

#### 2.2.1. Light Treatment

At 60 d of growth, *L. ruthenicum* seedlings of a consistent size were selected and their leaves were detached. The detached leaves were cut into small segments (~1 cm) and placed on the surface of solid MS medium with about 10 segments per plate. Plates were then incubated under one of the following conditions, each at 5000 lx and 50 W: white light (WL) (standard growth chamber bulb); BL (450 nm); red light (RL) (650 nm); or far-red light (FRL) (730 nm). The leaves were cultured at 25 °C under a 16/8 h light/dark photoperiod for 7 d prior to further analysis. There were 3 plates per light treatment. 

#### 2.2.2. Sucrose Treatment

At 60 d of growth, *L. ruthenicum* seedlings of a consistent size were selected and their leaves were detached. Detached leaves were cut into small segments (~1 cm) and placed on the surface of solid MS medium containing one of the following sucrose concentrations: 0, 100, 200, 300, 400, 500, 600, or 700 mM. The leaves were cultured at 25 °C under either white light (control) or blue light (450 nm) with a 16/8 h light/dark photoperiod for 7 d prior to further analysis. There were 3 plates per sucrose treatment.

### 2.3. Determination of Anthocyanidin Contents by HPLC

Samples treated with different light conditions and sucrose concentrations as described above were collected for anthocyanin quantification. The total anthocyanin content was first measured using the pH difference method and expressed in mg·g^−1^ of fresh weight (FW). For this method, anthocyanins were extracted in 80% ethanol with 1% hydrochloric acid and the total anthocyanin content was calculated as previous described [19]. 

The abundances of specific anthocyanin compounds were then quantified through high-performance liquid chromatography (HPLC)–tandem mass spectrometry (MS/MS). Fresh leaf samples were freeze-dried, ground to powder in a grinder (MM 400, Retsch) at 30 Hz for 1.5 min, and then stored at −80 °C until further analysis. For each sample, 50 mg of powder was extracted in 0.5 mL of 500:500:1 methanol:water:hydrochloric acid (*v*/*v*/*v*). The extracts were vortexed for 5 min and then treated using ultrasonication for 5 min. Samples were centrifuged at 12,000× *g* at 4 °C for 3 min and the supernatant was removed. For each sample, the residue was then re-extracted once by repeating the extraction steps. The supernatants were collected and filtrated through a membrane with a 0.22 μm pore size (Anpel Laboratory Technologies, Shanghai, China). The abundances of specific anthocyanin compounds were detected using MetWare (http://www.metware.cn/, accessed on 18 April 2023) based on the AB Sciex QTRAP 6500 LC-MS/MS platform (AB Sciex, Framingham, MA, USA) using an ACQUITY BEH C18 column (1.7 µm, 2.1 mm × 100 mm) (Waters, Milford, MA, USA). Solvent A was 0.1% formic acid in water and Solvent B was 0.1% formic acid in methanol. The gradient program was as follows: 19:1 Solvent A:B at 0 min; 1:1 Solvent A:B at 6 min; 1:19 Solvent A:B from 12 to 14 min; and 19:1 Solvent A:B from 14 to 16 min. The flow rate was 0.35 mL/min, the column temperature was 40 °C, and the injection volume was 2 μL. The electrospray ionization (ESI)-MS/MS conditions were as follows: ion source, ESI+; source temperature, 550 °C; ion spray (IS) voltage, 5500 V; curtain gas pressure, 35 psi. Peaks were detected and quantified using MetWare (http://www.metware.cn/, accessed on 18 April 2023).

### 2.4. RNA-Seq

The detached *L. ruthenicum* leaves treated with BL or WL and with 0 mM or 500 mM sucrose were selected as the experimental material for transcriptome analysis. Total RNA was extracted from the cultured leaf samples using the RNAprep Pure Plant Kit (Tiangen, Beijing, China) following the manufacturer’s instructions. The RNA concentration and purity were measured on a NanoDrop 2000 (Thermo Fisher Scientific, Wilmington, DE, USA). RNA integrity was assessed using the RNA Nano 6000 Assay Kit with the Agilent Bioanalyzer 2100 system (Agilent Technologies, Santa Clara, CA, USA). Sequencing libraries were generated using the NEBNext Ultra RNA Library Prep Kit for Illumina (New England Biolabs, Ipswich, MA, USA) following the manufacturer’s instructions. Index codes were added to identify each sample. Clean reads were aligned to the *L. ruthenicum* reference genome using Hisat2. Successfully aligned sequences were assembled and expression levels calculated using StringTie software to establish a transcriptome library.

### 2.5. Differentially Expressed Gene Analysis 

DEGs between samples were identified using the “DESeq2” R package (v1.16.1). The thresholds were false discovery rate (FDR)-adjusted *p* < 0.05 and |log2(fold change [FC])| ≥ 1. DEG expression patterns were displayed as heat maps, which were generated in R software. Enrichment analyses were conducted in the DEG sets using Gene Ontology (GO) annotation terms and Kyoto Encyclopedia of Genes and Genomes (KEGG) biochemical pathways with the “clusterProfiler” package in R, correcting for gene length bias. GO terms with corrected *p*-values < 0.05 were considered significantly enriched.

### 2.6. Cis-Acting Element Analysis

The promoter regions (defined as the 2000 bp regions upstream of the translation start sites) of key genes suspected to be responsive to light or sucrose were analyzed to identify putative cis-acting elements. Each promoter region was analyzed using the tool on the PlantCARE website (https://bioinformatics.psb.ugent.be/webtools/plantcare/html/, accessed on 26 June 2023).

### 2.7. Quantitative Reverse Transcription (qRT)–PCR Analysis

Primers for qRT-PCR (Table S1) were designed using the Integrated DNA Technologies (IDT) website. qRT-PCR was performed on an ABI system using the SuperReal fluorescence quantitative premix reagent (SYBR Green) kit (Tiangen, Beijing, China). Gene expression was normalized through the 2^−ΔΔCt^ method using β-actin as the internal control. 

### 2.8. Statistical Analysis

Statistically significant differences between treatment groups were assessed through analysis of variance (ANOVA) in SPSS v24.0 for Windows (SPSS Inc., Chicago, IL, USA) and post hoc Tukey’s test. Values were considered statistically significantly different at *p* ≤ 0.05.

## 3. Results

### 3.1. Effects of Light and Sucrose Treatments on Total Anthocyanin Content in L. ruthenicum Leaves

**Figure 1:**
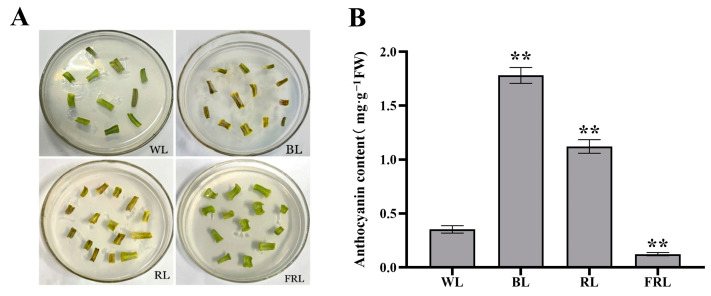
(**A**) Effects of light quality on detached *L. ruthenicum* leaf color. Leaves were cultured for 7 d under white light (WL), blue light (BL), red light (RL), or far-red light (FRL). (**B**) Quantification of anthocyanin content per gram of detached *L. ruthenicum* leaf tissue. Leaves were treated as described in (**A**). Error bars represent standard error from three biological replicates. ** *p* < 0.01 (analysis of variance with post hoc Tukey’s test).

As shown in Figure 1, the light quality treatments had significantly different effects on the coloration of *L. ruthenicum* leaves and total anthocyanin content. Compared with WL-treated leaves, those treated with BL showed large areas of dark purple coloration. Some leaves treated with RL turned dark purple, whereas those treated with FRL showed no color change or were a more intense green compared to WL-treated leaves. Thus, only the BL and RL treatments induced anthocyanin-related coloration in the detached *L. ruthenicum* leaves (Figure 1A). Consistent with this visual analysis, the total anthocyanin contents were significantly higher in BL-treated and RL-treated leaves compared to WL-treated leaves. BL-treated leaves had the highest total anthocyanin content (1.88 mg·g^−1^ FW), followed by RL-treated leaves (1.12 mg·g^−1^ FW), WL-treated leaves (0.35 mg·g^−1^ FW), and then FRL-treated leaves (0.10 mg·g^−1^ FW) (Figure 1B).

**Figure 2:**
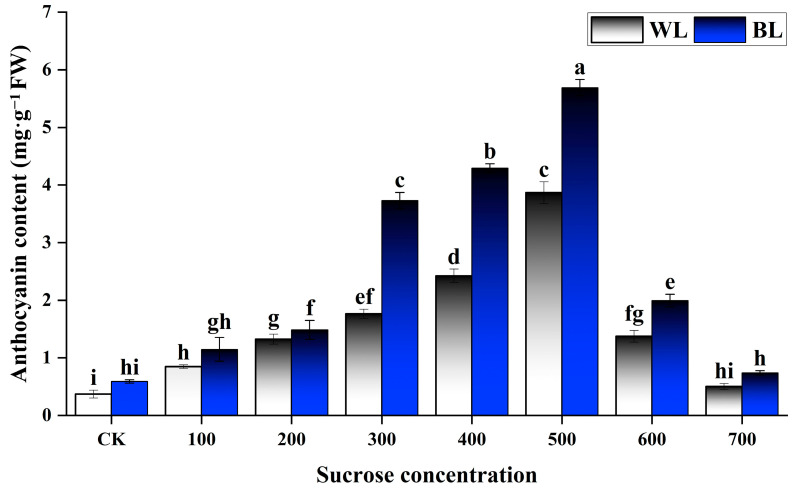
Effects of sucrose and blue light (BL) treatment on anthocyanin content in detached *L. ruthenicum* leaves. Sucrose concentrations are given in mM. CK, control (0 mM sucrose); WL, white light. Error bars represent standard error from three biological replicates. Lowercase letters above each bar represent significant differences at *p* < 0.05 (analysis of variance and post hoc Tukey’s test).

Among sucrose-treated leaves cultured under either WL or BL, low and moderate sucrose levels were associated with a steadily increasing anthocyanin content, whereas the anthocyanin content decreased again at the highest sucrose concentrations (Figure 2). The anthocyanin content peaked in leaves treated with 500 mM sucrose, which were a purple-black color; in this treatment group, leaves cultured under WL and BL contained 3.87 and 5.69 mg·g^−1^ FW anthocyanins, respectively (Figure 2). The 500 mM treatment was therefore selected for further experiments involving sucrose. At every sucrose concentration, leaves cultured under BL had a significantly higher total anthocyanin content than leaves cultured under WL, suggesting that BL treatment had significant effects on anthocyanin accumulation (*p* < 0.05). Under each light treatment, there were significant differences in anthocyanin content between sucrose concentrations (*p* < 0.05), demonstrating that anthocyanin production was sucrose-inducible.

### 3.2. Effects of Light and Sucrose Treatments on Specific Anthocyanins in L. ruthenicum Leaves

The abundances of specific anthocyanins were next assessed via HPLC/MS-MS in detached *L. ruthenicum* leaves treated with BL or WL and with 0 mM or 500 mM sucrose. A total of 40 anthocyanins and derivatives (namely one pro-anthocyanidin and three flavonoids) were identified (Table S1). Overall, the total anthocyanin content was highest in leaves cultured under BL with 500 mM sucrose (BL[+S]), followed by those cultured under WL with 500 mM sucrose (WL[+S]), BL without sucrose (BL[−S]), and finally WL without sucrose (WL[−S]). These results were consistent with the results discussed above. 

**Figure 3:**
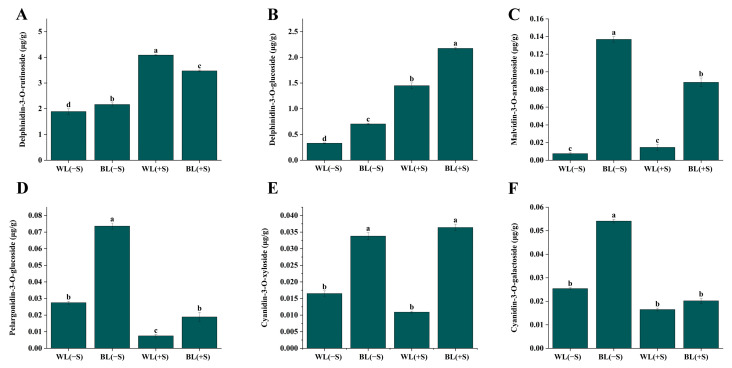
Differences in specific anthocyanin contents among detached *L. ruthenicum* leaves in response to blue light (BL) and sucrose treatments. WL(−S), plants treated with white light and no sucrose; BL(−S), plants treated with BL and no sucrose; WL(+S), plants treated with white light and 500 mM sucrose; BL(+S), plants treated with BL and 500 mM sucrose. (**A**) delphinidin-3-O-rutinoside, (**B**) delphinidin-3-O-glucoside, (**C**) malvidin-3-O-arabinoside, (**D**) pelargonidin-3-O-glucoside, (**E**) cyanidin-3-O-xyloside, (**F**) cyanidin-3-O-galactoside. Error bars represent standard error from three biological replicates. Lowercase letters above each bar represent significant differences at *p* < 0.05 level (analysis of variance and post hoc Tukey’s test).

Some representative substances with high accumulation levels in detached *L. ruthenicum* leaves were delphinidin-3-O-rutinoside, delphinidin-3-O-glucoside, malvidin-3-O-arabinoside, pelargonidin-3-O-glucoside, cyanidin-3-O-rutinoside, cyanidin-3-O-xyloside, and cyanidin-3-O-galactoside (Table S2), in which the most abundant anthocyanins in detached *L. ruthenicum* leaves were delphinidin and its derivatives. Among them, delphinidin-3-O-rutinoside and delphinidin-3-O-glucoside levels were significantly increased in the BL(−S), BL(+S), and WL(+S) leaves compared to WL(−S) leaves (*p* < 0.05) (Figure 3), and the content also accounted for a large proportion in the total anthocyanin. Malvidin-3-O-arabinoside levels were significantly higher in BL(−S) than WL(+S) leaves, and pelargonidin-3-O-glucoside and cyanidin-3-O-galactoside contents were significantly increased in BL(−S) leaves but significantly decreased in BL(+S) and WL(+S) leaves, compared to WL(−S) leaves (*p* < 0.05). Cyanidin-3-O-rutinoside only exhibited a significant accumulation in BL(+S) leaves. We therefore inferred that delphinidin-3-O-rutinoside and delphinidin-3-O-glucoside production were differentially regulated by the BL(−S), BL(+S), and WL(+S) treatments.

### 3.3. Transcriptome Sequencing and DEG Analysis

RNA-seq was carried out to elucidate the molecular mechanisms underlying BL- and sucrose-induced regulation of anthocyanin biosynthesis in detached *L. ruthenicum* leaves. A total of 82.72 Gb of clean reads were obtained, averaging 6.05 Gb per sample. The percentages of Q30 bases in each sample were ≥92.55% (Table 1). Comparison to the reference genome yielded between 3796.3 × 10^4^ and 5378.3 × 10^4^ aligned sequences per sample, with mapping rates between 93.19% and 94.20% (Table S3). Within-group Pearson’s correlation coefficients were >0.95, and the correlation coefficients were even higher between groups. Expression levels of individual genes were calculated in fragments per kilobase of transcript per million mapped reads (FPKM); principal component analysis (PCA) based on the FPKM values of all genes in each sample showed both significant differences between groups and good intragroup reproducibility.

**Figure 4:**
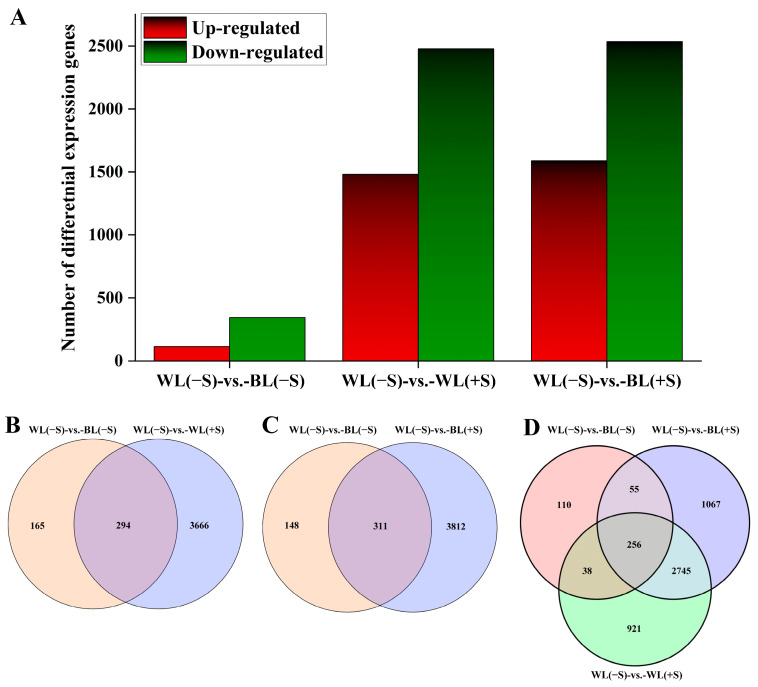
Differentially expressed gene analyses. (**A**) The number of DEGs in three comparison groups: leaves treated with white light and no sucrose (WL[−S]) compared to leaves treated with blue light and no sucrose (BL[−S]); WL(−S) compared to leaves treated with white light and 500 mM sucrose (WL[+S]); and WL(−S) compared to leaves treated with blue light and 500 mM sucrose (BL[+S]). (**B**–**D**) Unique and overlapping DEGs between the comparisons of WL(−S) with (**B**) BL(−S) and WL(+S), (**C**) BL(−S) and BL(+S), and (**D**) BL(−S), WL(+S), and BL(+S).

Using an FDR-adjusted *p*-value threshold of 0.05 and a |log2(FC)| threshold of 1, selected pairwise comparisons among the WL(−S), BL(−S), WL(+S), and BL(+S) samples yielded a total of 8542 DEGs (Figure 4A). There were 459 DEGs (115 up-regulated and 344 down-regulated) in the comparison of WL(−S) with BL(−S) (Table S4); 3960 DEGs (1482 up-regulated and 2478 down-regulated) in the comparison of WL(−S) to WL(+S) (Table S5); and the largest number of 4123 DEGs (1589 up-regulated and 2534 down-regulated) in WL(−S) compared to BL(+S) (Table S6). These three comparisons were designed to identify differences in gene expression associated with differences only in light quality, only in the sucrose concentration, and in both light quality and sucrose concentration, respectively. There were 294 DEGs in common across the WL(−S)-vs.-BL(−S) to WL(−S)-vs.-WL(+S) (Figure 4B), and 311 DEGs in common across the WL(−S)-vs.-BL(−S) to WL(−S)-vs.-BL(+S) (Figure 4C). There were 256 DEGs in common across the three comparisons and 110, 1067, and 921 unique DEGs in the comparisons of WL(−S)-vs.-BL(−S), WL(−S)-vs.-BL(+S), and WL(−S)-vs.-WL(+S), respectively (Figure 4D).

### 3.4. Functional Enrichment and Anthocyanin Structural Gene Analyses

To understand the functions of DEGs in each of the three comparison groups, we conducted GO annotation enrichment analysis. Significantly enriched GO terms in the three sets of DEGs included the biological process terms “metabolic process”, “cellular process”, “biological regulation”, and “response to stimulus”. Enriched molecular function terms included “catalytic activity”, “binding”, “transporter activity”, and “nucleic acid binding transcription factor activity”, and enriched cellular component terms included “cell part”, “cell”, “membrane”, “membrane part”, and “organelle” (Figure S1).

KEGG pathway enrichment analysis was carried out to gain further insights into the biochemical pathways to which the DEGs belonged (Figure S2). In the comparison of WL(−S)-vs.-BL(−S), enriched pathways included “plant hormone signal transduction”, “photosynthesis-antenna proteins”, “plant circadian rhythm”, “MAPK signaling pathway-plant”, “alpha-linolenic acid metabolism”, and “ABC transporters”. The terms “plant hormone signal transduction”, “MAPK signaling pathway-plant”, “starch and sucrose metabolism”, and “phenylpropanoid biosynthesis” were significantly enriched in the comparisons of WL(−S)-vs.-WL(+S) and WL(−S)-vs.-BL(+S). We selected several pathways associated with light responses, sugar signaling, and anthocyanin synthesis for further analysis because they are important components of environmental adaptation and specialized metabolite biosynthesis: “plant hormone signal transduction”, “plant circadian rhythm”, “starch and sucrose metabolism”, “phenylpropanoid biosynthesis”, “flavonoid biosynthesis”, and “anthocyanin biosynthesis”. Based on the selected DEGs enriched by the KEGG pathway combined with enriched GO functional annotations, we screened DEGs related to light responsiveness, MAPK signaling, and anthocyanin biosynthesis. *CRY1*, *PHYTOCHROME INTERACTING FACTOR 3* (*PIF3*), *COP1*, and *HY5* were annotated as members of the plant circadian rhythm pathway; the *PYR/PYL* abscisic acid (ABA) receptor family, *PROTEIN PHOSPHATASE 2C* (*PP2C*), *SNF-RELATED SERINE/THREONINE PROTEIN KINASE 2* (*SnRK2*), and *ABSCISIC ACID INSENSITIVE 5* (*ABI5*) were annotated as members of the plant MAPK signaling pathway; and anthocyanin-biosynthesis-related genes including *CHS*, *CHI*, *FLS*, *F3H*, *F3′H*, *F3′5′H*, *DFR*, *ANS*, and *UFGT* were annotated as members of the flavonoid biosynthesis and anthocyanin biosynthesis pathways.

**Figure 5:**
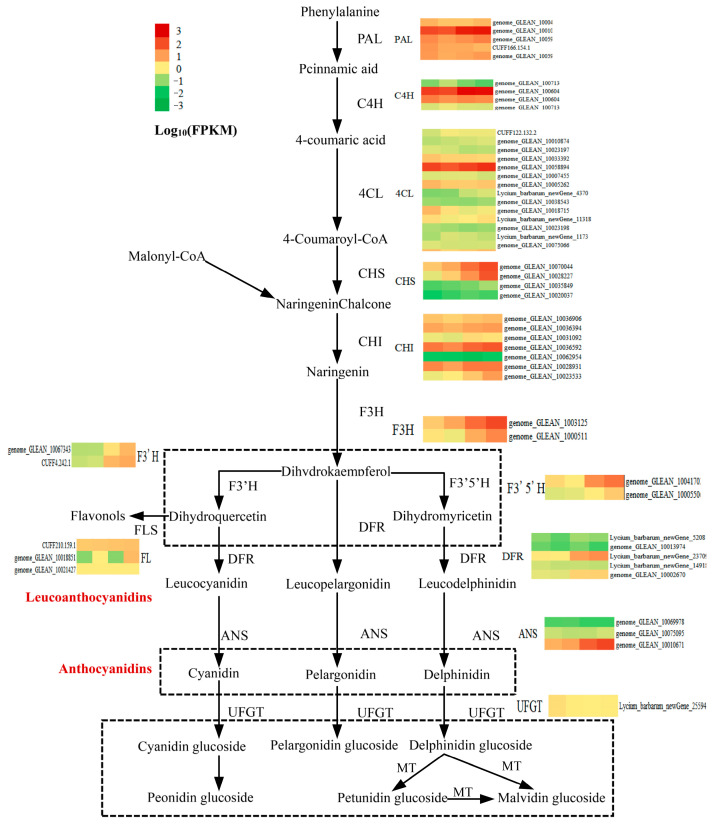
Expression patterns of genes in the anthocyanin synthesis pathway in leaves treated with blue light (BL) and/or sucrose. Gene expression values are shown in log_10_ (fragments per kilobase of transcript per million mapped reads (FPKM)). All heat maps show expression levels for the indicated genes in the following order from left to right: leaves treated with white light and no sucrose; leaves treated with BL and no sucrose; leaves treated with white light and 500 mM sucrose; and leaves treated with BL and 500 mM sucrose.

As expected, one 4CL (*Lycium_barbarum_new Gene_4370*) gene was up-regulated through sucrose treatment and BL(+S) treatment, and was enriched in the “phenylpropanoid biosynthesis” pathway. Some structural genes (*CHS*, *CHI*, *FLS*, *F3H*, *F3′H*, *F3′5′H*, *DFR,* and *ANS*) involved in the biosynthesis of flavonoids, flavonols, and anthocyanins were also significantly up-regulated, and enriched in the “flavonoid biosynthesis” pathway under WL(+S) treatment and BL(+S) treatment (Figure 5). CHS (*genome_GLEAN_10028227*) was up-regulated by 2.24-fold under BL treatment, 3.45-fold under sucrose treatment, and 4.72-fold under BL(+S) treatment. *CHI* (*genome_GLEAN_10023533*) was up-regulated by 1.96- and 2.84-fold under WL(+S) treatment and BL(+S) treatment, respectively. FLS (*genome_GLEAN_10018851*) was significantly up-regulated by 6.37- and 7.76-fold under the BL(−S) treatment and BL(+S) treatment, respectively. *F3H* (*genome_GLEAN_10031253*) was up-regulated by 1.89- and 2.47-fold under WL(+S) treatment and BL(+S) treatment, respectively. *F3′H* (*genome_GLEAN_10067343*) was up-regulated by 4.30- and 4.70-fold under WL(+S) treatment and BL(+S) treatment, respectively. The expression of *F3′5′H* (*genome_GLEAN_10041702*) was increased by 1.38- and 1.75-fold under WL(+S) treatment and BL(+S) treatment, respectively. *DFR* (*Lycium_barbarum_newGene _23709*) was up-regulated by 1.71-fold under BL(+S) treatment. *ANS* (*genome_GLEAN_10010671*) was up-regulated by 1.88-fold under blue-BL(+S) treatment. 

### 3.5. Anthocyanin-Related Genes Induced by BL and/or Sucrose Treatment 

The RNA-seq data clearly showed that some anthocyanin biosynthesis genes were responsive to light quality. We next used co-expression analysis to identify potential upstream light-responsive genes associated with anthocyanin synthesis. Analysis of photoreceptors indicated that the cryptochrome gene *CRY1* (*genome_GLEAN_10072304*) was significantly down-regulated among detached *L. ruthenicum* leaves cultured under BL compared to those cultured under white light. Because the white fluorescent lamp emitted red, blue, and green light, changes in *CRY1* expression were clearly caused by exposure to only BL. Analysis of other known genes in the light signaling pathway revealed that *PIF3* (*genome_GLEAN_10075126*) and *COP1* were down-regulated under BL, whereas *HY5* (*genome_GLEAN_10056214*) was up-regulated. Thus, analysis of anthocyanin contents and the expression levels of anthocyanin structural genes, photoreceptors, and transcription factors suggested that anthocyanin accumulation in detached *L. ruthenicum* leaves was likely caused by BL signaling.

We next analyzed genes potentially associated with anthocyanin biosynthesis in response to sucrose-induced osmotic stress. Two *CLAVATA 1* genes (*genome_GLEAN_10051147* and *genome_GLEAN_10040926*) were down-regulated, which led to the overexpression of *NCED1* genes (*genome_GLEAN_10065758* and *genome_GLEAN_10001057*). Expression analysis indicated that increased NCED1 expression may be associated with ABA biosynthesis. Furthermore, two *PYR/PYL1* genes (*genome_GLEAN_10053483* and *genome_GLEAN_10066050*) were down-regulated, two *PP2Cs* (*genome_GLEAN_10053585* and *genome_GLEAN_10020648*) were significantly up-regulated, and *SnRK2* (*genome_GLEAN_10067681*) was significantly up-regulated in protein phosphatase and protein kinase. SnRK2 promotes the expression of downstream anthocyanin synthesis genes by regulating the phosphorylation of ABI5 (*genome_GLEAN_10061387*), which was also significantly up-regulated. Importantly, both the BL and sucrose treatments induced *BBX* (*genome_GLEAN_10076597*), which induces or interacts with both HY5 and ABI5 to regulate BL-mediated ABA signal transduction. The results thus suggested that these three transcription factors formed a positive feedback system at the transcriptional level that was involved in regulating anthocyanin synthesis under BL and sucrose-induced osmotic stress conditions.

### 3.6. Anthocyanin-Related Transcription Factors Induced by BL and/or Sucrose Treatment 

**Figure 6:**
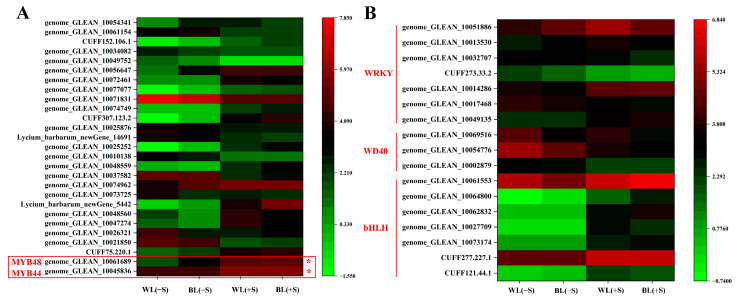
Expression levels of selected transcription factors that were differentially expressed in response to blue light and/or sucrose-induced osmotic stress. Transcription factors in the (**A**) MYB and (**B**) bHLH, WD40, and WRKY families were clustered based on similarities in expression patterns. Gene expression levels are indicated by color corresponding to log_2_ (fragments per kilobase of transcript per million mapped reads (FPKM) + 1) values. Red boxes with ‘*’ indicated the candidate MYB TFs.

We next sought to more comprehensively identify the specific transcription factors that regulated anthocyanin structural genes in response to BL and sucrose treatment. There were twenty-seven MYBs, seven bHLHs, three WD40s, and seven WRKYs that were differentially expressed in response to BL and/or sucrose treatment. In comparison to WL(−S), 17 MYBs were up-regulated and 10 were down-regulated in WL(+S), and 13 MYBs were up-regulated and 8 were down-regulated in BL(+S). The *R2R3-MYB* genes *LrMYB44* (*genome_GLEAN_10045836*) and *LrMYB48* (*genome_GLEAN_10061689*) were significantly up-regulated in both WL(+S) and BL(+S) compared to WL(−S) (Figure 6A). *bHLH35* (*CUFF277.227.1*) and *bHLH137* (*genome_GLEAN_10061553*) were also significantly up-regulated in the WL(+S) and BL(+S) treatments (Figure 6B). 

Induction of *LrMYB44* and *LrMYB48* through the BL and sucrose treatments suggested that these two transcription factors may have been involved in stress responses. To determine the potential functions of these genes, the promoter regions were examined for the presence of putative *cis*-acting elements. The *LrMYB44* promoter contained multiple abiotic stress *cis*-elements (Table 2), such as light-responsive elements (a GT1-motif, Box 4, G-box, TCT-motif, GA-motif, and an MRE), ABA response elements (ABREs), elements involved in the gibberellin response (e.g., a P-box), and drought-inducible MYB binding sites (MBSs). Abiotic-stress-related *cis*-elements were also found in the *LrMYB48* promoter (Table 3). These included light-response elements (a G-box, LAMP-element, and TCT-motif), elements involved in low-temperature responses (LTRs), MBSs, *cis*-acting regulatory elements involved in methyl jasmonate reactivity (a CGTCA-motif and TGACG-motif), ABREs, and elements involved in defense and stress responses (TC-rich repeats).

To establish potential regulatory factors of *LrMYB44* and *LrMYB48*, we calculated correlation coefficients between expression levels of the two genes and of *HY5* and *ABI5*. Both genes were significantly positively correlated with *HY5* and *ABI5* (R^2^ = 0.9582 and 0.9967, respectively; *p* < 0.01). This suggested that *LrMYB44* and *LrMYB48* could be regulated by the direct binding of HY5 and AB15 to the G-box elements and ABREs, respectively, in their promoters, enhancing anthocyanin synthesis in detached *L. ruthenicum* leaves in response to BL and sucrose-induced osmotic stress.

**Figure 7:**
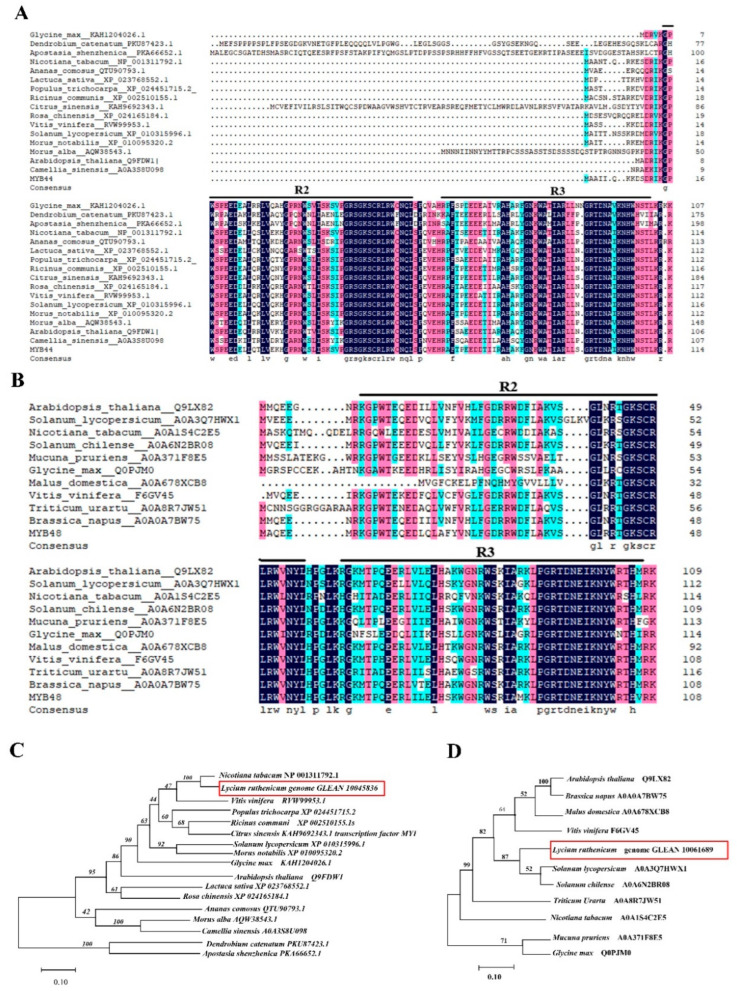
Phylogenetic analysis of candidate transcription factors regulating anthocyanin biosynthesis in *L. ruthenicum*. (**A**,**B**) Amino acid sequence alignments of (**A**) LrMYB44 and (**B**) LrMYB48 with homologs in other plant species. (**C**,**D**) Phylogenetic trees showing the relationships of (**C**) LrMYB44 and (**D**) LrMYB48 with homologs in other plant species.

To further establish the functions of LrMYB44 and LrMYB48, we performed conserved domain analysis. LrMYB44 contained two conserved MYB-DNA binding domains, which were located at residues 14–60 and 66–111 (Figure 7A). LrMYB48 also had two conserved domains, located at residues 8–55 and 61–106 (Figure 7B). Comparing the amino acid sequences of LrMYB44 and LrMYB48 with paralogs showed that LrMYB44 and LrMYB48 contained two evolutionarily conserved amino acid sequences, specifically MYB repeat DNA-binding sequences at the C-terminal. A phylogenetic tree was then constructed to analyze the evolutionary relationships among LrMYB44, LrMYB48, and homologs in other species. *LrMYB44* was most closely related to a homolog in *Nicotiana tabacum* (100% similarity) (Figure 7C). A transient expression assay in tobacco indicated that *NtMYB44* may be involved in the light-induced anthocyanin biosynthesis pathway, suggesting that *LrMYB44* regulated genes related to anthocyanin biosynthesis. *LrMYB48* was most closely related to homologs in tomato and potato (Figure 7D). 

Based on the functional domains present in LrMYB44 and LrMYB48 and their suspected roles in anthocyanin biosynthesis, we next examined anthocyanin structural genes for evidence of regulation by LrMYB44 or LrMYB48. Indeed, the promoter regions of *F3′H* (Table S7), *F3′5′H* (Table S8), and *ANS* (Table S9) all contained MYB and MYC binding elements. We therefore hypothesized that the candidate transcription factors LrMYB44 and LrMYB48 may have regulated *F3′H*, *F3′5′H*, and *ANS* expression through direct binding to the promoter regions. This hypothesis was supported by significant positive correlations between *LrMYB44* and *LrMYB48* expression levels and those of *CHI*, *F3H*, *F3′H*, *F3′5′H*, and *ANS*. We also identified numerous relevant *cis*-elements in the *F3′H*, *F3′5′H*, and *ANS* promoters, such as ABREs and elements involved in light responsiveness (G-box elements). Overall, these results indicated that anthocyanin synthesis was promoted in response to BL and/or sucrose via the up-regulation of MYB transcription factors. Specifically, the MYBs LrMYB44 and LrMYB48 likely regulated anthocyanin synthesis and accumulation in detached *L. ruthenicum* leaves under BL conditions and/or sucrose-induced osmotic stress treatment.

### 3.7. qRT-PCR Validation of Candidate Genes

**Figure 8:**
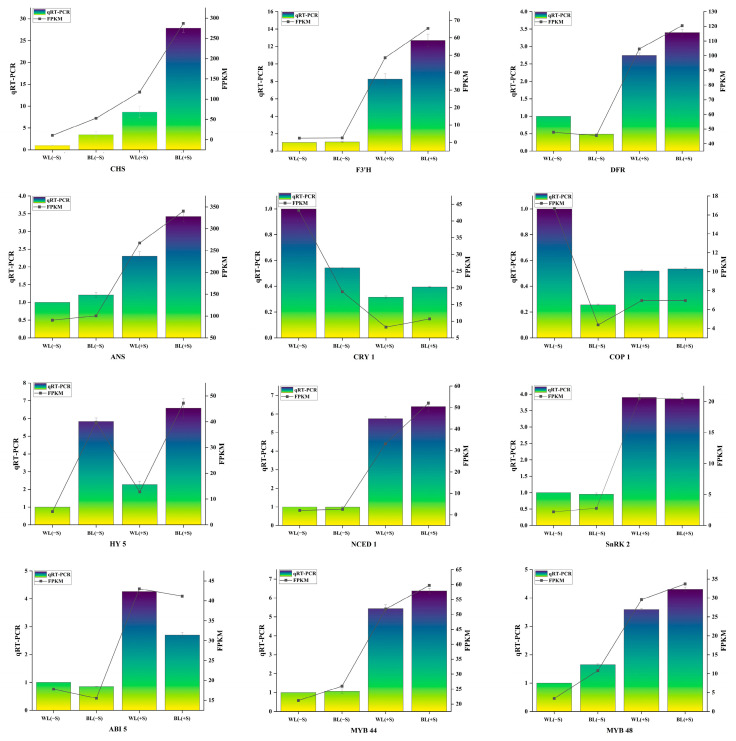
Validation of selected candidate gene expression levels with quantitative reverse transcription PCR.

To validate the RNA-seq results, several genes were quantified through qRT-PCR: six genes related to anthocyanin synthesis (*CHS*, *DFR*, *F3′H*, and *ANS*), two transcription factors (*LrMYB44* and *LrMYB48*), four genes involved in light signaling and transmission (*CRY1*, *COP1*, and *HY5*), and four genes in the plant MAPK signaling pathway (*NCED1*, *SnRK2,* and *ABI5)*. *CHS* and *FLS* were significantly up-regulated under BL and sucrose treatments (Figure 8). *DFR*, *ANS*, *LrMYB44*, and *LrMYB48* were significantly up-regulated in response to sucrose treatment but not BL. *CRY1* and *COP1* were down-regulated under both the BL(−S) and BL(+S) treatments. *HY5* expression was increased under BL compared to WL conditions. *NCED1*, *SnRK2*, and *ABI5* were significantly up-regulated in response to sucrose treatment. Thus, the qRT-PCR results were fully consistent with and validated the reliability of the RNA-seq data.

## 4. Discussion

Anthocyanins are key specialized plant metabolites with numerous important roles in development, reproduction, and stress responses. Anthocyanin biosynthesis is a branch of the flavonoid biosynthesis pathway and is specifically up-regulated by a variety of conditions, including stressors. These compounds are critical indicators of plant quality in *L. ruthenicum* due to their beneficial health effects; although *L. ruthenicum* naturally produces high volumes of anthocyanins, the associated mechanisms in response to light quality and sucrose treatment remain unclear. 

Light is an important environmental factor that affects plant growth and development throughout the life cycle, acting as both a source of energy and a signal for development [20]. Specific light conditions or qualities can induce anthocyanin production. BL in particular is known for triggering anthocyanin production in plants such as strawberry [21], pear [12], eggplant [22], *Petuniahybrida Vilm* [23], *Myrica rubra* [24], and purple pepper [25]. This occurs through increases in the activities of enzymes associated with anthocyanin biosynthesis. Such responses are mediated by CRYs, which function as BL sensors and trigger a variety of downstream reactions [26,27].

In addition to specific types of light, anthocyanins are commonly induced by osmotic stress conditions, which is beneficial to the plant cells because anthocyanins could be used directly as osmotic adjustment substances, and also effectively maintain and improve the active oxygen scavenging abilities [20]. Osmotic stress responses, including anthocyanin biosynthesis, are mediated by plant hormones, which are essential compounds that regulate a variety of stress responses [28]. ABA in particular is induced by osmotic stress conditions, among other abiotic stressors, and up-regulates anthocyanin production. For example, sucrose treatment increases ABA synthesis in the fruits, which subsequently leads to increases in sugar and anthocyanin levels [29]. In species such as rice [30], *Fagopyrum tataricum*, and apple [31], ABA treatment induces anthocyanin accumulation. 

CRY proteins sense blue light and transduce the signal to downstream proteins such as COP1. In response to BL, COP1 is expressed by the cytosol, where its activity is repressed by photoreceptors; this leads to activation of photomorphogenesis and anthocyanin biosynthesis [32]. A previous study revealed that COP1 could degrade HY5, which acts as a core regulator of the light signaling pathway and plays important roles in anthocyanin biosynthesis regulation. In *Arabidopsis*, CRYs interact with COP1 under BL conditions, decreasing the COP1-Ub-mediated degradation of HY5 and thus increasing anthocyanin accumulation. In tomato, plants overexpressing *CRY1a* show increased anthocyanin accumulation, and 3 h of BL treatment decreases *SlCOP1* transcription while increasing *SlHY5* expression [14]. This indicates that BL initially affects *SlCRY1a* and *SlCOP1*, which induce further responses via interactions with *SlHY5*. 

HY5 is the first transcription factor that was found to be involved in photomorphogenesis, and it plays a key regulatory role in anthocyanin biosynthesis [33]. This regulation occurs via direct binding to the promoters of anthocyanin-biosynthesis-related genes. HY5-regulated genes include *CHS* and *DFR* in eggplant [22], and *CHS* in *Arabidopsis* [34]. In apple, MdHY5 increases anthocyanin content by directly activating *MdMYB10* expression [35]. In peach, *PpHY5* is up-regulated through UV-A and UV-B treatment; PpHY5 enhances its own transcription and expression of the downstream anthocyanin biosynthesis genes *PpCHS1*, *PpCHS2*, *PpDFR1*, and *PpMYB10.1* through interactions with the E-boxes in their promoters [36]. In pear, HY5 binding to the G-Box in the promoter region of *MYB10* induces transcription of the gene and promotes anthocyanin biosynthesis in the fruit [37]. Similarly, in the red pear “Yunhongli No. 1”, HY5 directly binds the G-box element in the *MYB10* promoter to enhance its expression, increasing anthocyanin accumulation in the epidermis [38].

High levels of sucrose up-regulate *NCED*, which encodes a 9-cis-epoxycarotenoid dioxygenase that catalyzes the rate-limiting step in ABA biosynthesis. ABA binds to PYR/PYL, which activates PP2C and SnRK2, and then PP2C and SnRK2 significantly up-regulate ABI5, a key bZIP transcription factor that mediates activation of anthocyanin structural genes through interactions with other transcription factors. For example, in apple, MdABI5 positively regulates ABA-induced anthocyanin synthesis by regulating the MdbHLH3-MdMYB1 complex [31]. In *Arabidopsis*, AtABI5-4 regulates anthocyanin synthesis via formation of protein complexes with AtTTG1, AtTT8, and AtMYB75 [39,40].

In the present study, we sought to elucidate the mechanism by which BL and sucrose-induced osmotic stress regulated anthocyanin accumulation in *L. ruthenicum*. The initial analysis showed that anthocyanin levels first increased and then decreased along with increasing sucrose levels under both BL and WL conditions, peaking at 500 mM sucrose under both light qualities. Furthermore, BL treatment was associated with significantly higher anthocyanin levels than WL across sucrose concentrations. This confirmed that anthocyanins were induced by the BL and osmotic stress treatments as expected. Transcriptomic analysis showed that the anthocyanin structural genes *CHS* and *FLS* were up-regulated under BL treatment; *CHI*, *CHS*, *F3H*, *F3′H*, and *F3′5′H* were significantly up-regulated in response to sucrose-induced osmotic stress; and *CHI*, *CHS*, *FLS*, *F3H*, *F3′H*, *F3′5′H*, *DFR*, and *ANS* were significantly up-regulated in leaves treated with both BL and sucrose. Moreover, anthocyanin biosynthesis was associated with up-regulation of transcription factors in the MYB, bHLH, and WD40 families. *LrMYB44* and *LrMYB48*, which were annotated as members of the anthocyanin biosynthesis pathway, were up-regulated in leaves treated with BL and sucrose. Analysis of the promoter regions of these two *MYB* genes revealed multiple light-response elements (e.g., G-boxes), drought-inducible MYB binding sites, and response elements induced by various plant hormones, such as ABA and methyl jasmonate. Together, these results indicated that *LrMYB44* and *LrMYB48* were likely involved in BL- and sucrose-induced anthocyanin biosynthesis in detached *L. ruthenicum* leaves.

Further analysis of the transcriptomic data suggested that sucrose-induced osmotic stress increased ABA accumulation by up-regulating *NCED1*, which ultimately increased the expression of *ABI5*. In leaves treated with high concentrations of sucrose, ABI5 might have interacted with MYBs to form protein complexes that regulated anthocyanin synthesis, promoting anthocyanin accumulation in response to stress. ABI5 could then regulate *LrMYB44* and *LrMYB48* through direct binding to ABREs in their promoters, and LrMYB44 and LrMYB48 could form protein complexes to induce anthocyanin accumulation, promoting osmotic stress tolerance. In addition, *HY5* expression was positively correlated with expression levels of *LrMYB44* and *LrMYB48*, indicating that HY5 may have bound to the G-boxes in the *LrMYB44* and *LrMYB48* promoters to activate their transcription. This is consistent with mechanisms previously identified in peach, pear, *Arabidopsis* [41], and apple [42]. 

**Figure 9:**
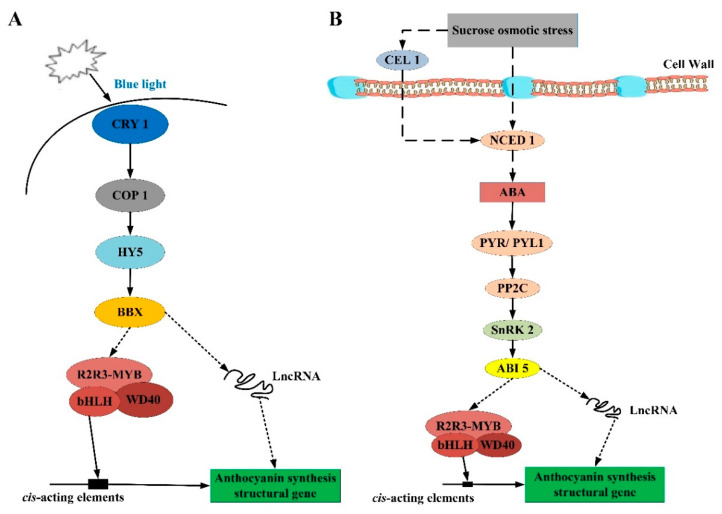
Proposed models showing regulation of anthocyanin synthesis in response to (**A**) blue light and (**B**) sucrose-induced osmotic stress.

Based on these results, we established comprehensive models of the mechanisms by which anthocyanin synthesis was likely regulated by BL (Figure 9A) and sucrose (Figure 9B). CRY1 functioned as a photoreceptor, perceiving blue light and activating downstream elements such as COP1, HY5, and BBX. BBX then induced anthocyanin synthesis by regulating long non-coding RNAs (lncRNAs) and members of the MBW complex, which activated the transcription of anthocyanin structural genes (Figure 9A). In contrast, sucrose-induced osmotic stress up-regulated *NCED1*, which led to ABA biosynthesis. The ABA-sensing module comprising PYR/PYL1 and PP2C transduced the signal to the ABA-dependent SnRK2, which activated ABI5. Like BBX, ABI5 then up-regulated anthocyanin structural genes through activation of the MBW complex and/or lncRNAs (Figure 9B). These models are consistent with the transcriptomic and qRT-PCR data generated here and with prior studies demonstrating similar mechanisms in other plant species. However, the specific functions of ABI5, BBX, and HY5 regarding how to interact with *LrMYB44* and *LrMYB4* to regulate anthocyanin biosynthesis in *L. ruthenicum* leaves remains to be further investigated and further studies are required to validate the hypothesized protein–protein and protein–DNA interactions.

## 5. Conclusions

We established here a co-regulatory mechanism by which *L. ruthenicum* likely responded to BL and sucrose-induced osmotic stress. This pathway included the anthocyanin structural genes *CHS*, *CHI*, *FLS*, *F3H*, *F3′H*, *F3′5′H*, *DFR*, *ANS*, and *UFGT*; the regulatory genes *LrMYB44*, *LrMYB48*, *bHLH35*, *bHLH137*, *RAP2-3*, and *ERF4*; the photoreceptor CRY1 and its downstream interactors PIF3, COP1, and HY5; and the MAPK signaling factors PYR/PYL, PP2C, SnRK2, and ABI5. The transcription factors HY5 and ABI5 were involved in the BL response through the light signal transduction pathway and in the sucrose-induced osmotic stress response through the ABA-dependent pathway, respectively. The overexpression of *BBX*, *HY5*, or *ABI5* enhanced the anthocyanin-related gene expression and anthocyanin accumulation. However, the functions of these genes in anthocyanin synthesis require further biological validation. In addition, to fully elucidate the mechanisms by which BL and sucrose induce anthocyanin synthesis, the expression profiles of detached *L. ruthenicum* leaves cultured under BL and sucrose-induced osmotic stress require investigation. Overall, this study provides valuable new insights into the mechanisms underlying the responses of *L. ruthenicum* to light quality and osmotic stress. Future experiments could leverage these findings to increase stress tolerance in this economically important plant.

## Figures and Tables

**Table 1:** Sequencing data quality statistics.

Samples	Clean Reads	Clean Bases (Gb)	Q20 (%)	Q30 (%)	GC Content (%)
W(−S)3d-1	24,159,442	7.23	97.65	93.61	42.86
W(−S)3d-2	21,728,052	6.50	98.00	94.39	42.96
W(−S)3d-3	20,227,658	6.05	97.83	94.03	42.75
B(−S)3d-1	28,569,810	8.55	98.07	94.62	43.35
B(−S)3d-2	21,464,668	6.42	97.94	94.31	42.81
B(−S)3d-3	21,453,863	6.4	97.88	94.21	42.91
W(+S)3d-1	21,418,743	4.41	97.61	93.47	42.78
W(+S)3d-2	22,778,765	6.81	97.27	92.55	43.01
W(+S)3d-3	26,353,973	7.88	97.92	94.20	42.95
B(+S)3d-1	24,735,098	7.40	98.04	94.55	42.76
B(+S)3d-2	22,170,577	6.64	98.04	94.47	42.67
B(+S)3d-3	21,398,823	6.40	97.86	94.09	42.60

Note: WL(−S), leaves treated with white light and no sucrose; BL(−S), leaves treated with blue light and no sucrose; WL(+S), leaves treated with white light and 500 mM sucrose; BL(+S), leaves treated with blue light and 500 mM sucrose. Numbers 1 through 3 at the end of the sample names indicate biological replicate samples.

**Table 2:** *Cis*-acting elements present in the *LrMYB44* promoter.

Key Cis-Acting Elements	Sequence	Number	Function
GT1-motif	GGTTAA	6	light responsive element
Box 4	ATTAAT	6	part of a conserved DNA module involved in light responsiveness
G-box	TACGTG	6	cis-acting regulatory element involved in light responsiveness
G-Box	CACGTT	6	cis-acting regulatory element involved in light responsiveness
TCT-motif	TCTTAC	6	part of a light responsive element
GA-motif	ATAGATAA	8	part of a light responsive element
MRE	AACCTAA	7	MYB binding site involved in light responsiveness
ABRE	ACGTG	5	cis-acting element involved in the abscisic acid responsiveness
P-box	CCTTTTG	7	gibberellin-responsive element
MBS	CAACTG	6	MYB binding site involved in drought-inducibility

**Table 3:** *Cis*-acting elements present in the *LrMYB48* promoter.

Key Cis-Acting Elements	Sequence	Number	Function
G-box	TACGTG	6	cis-acting regulatory element involved in light responsiveness
LAMP-element	CTTTATCA	8	part of a light responsive element
TCT-motif	TCTTAC	7	part of a light responsive element
LTR	CCGAAA	6	cis-acting element involved in low-temperature responsiveness
MBS	CAACTG	6	MYB binding site involved in drought-inducibility
CGTCA-motif	CGTCA	5	cis-acting regulatory element involved in the MeJA-responsiveness
TGACG-motif	TGACG	5	cis-acting regulatory element involved in the MeJA-responsiveness
ABRE	ACGTG	5	cis-acting element involved in the abscisic acid responsiveness
TC-rich repeats	GTTTTCTTAC	10	cis-acting element involved in defense and stress responsiveness

## Data Availability

The datasets generated or analyzed in the current study are available from the corresponding author on reasonable request. Data is not publicly available due to privacy or ethical restrictions.

## Supplementary Materials

The following supporting information can be downloaded at https://www.mdpi.com/article/10.3390/metabo13091004/s1, Table S1: Primers of the candidate genes for qRT-PCR; Table S2: The detailed information of 40 anthocyanins and derivatives identified in *L. ruthenicum* leaves; Table S3: Statistics of sequence alignment results between transcriptome data and reference genome; Table S4: Differentially expressed genes in the comparison of WL(−S) with BL(−S); Table S5: Differentially expressed genes in the comparison of WL(−S) to WL(+S); Table S6: Differentially expressed genes in the comparison of WL(−S) compared to BL(+S); Table S7: Cis-acting elements present in the F3′H promoter; Table S8: Cis-acting elements present in the F3′5′H promoter; Table S9: Cis-acting elements present in the ANS promoter; Figure S1: GO annotation enrichment analysis; Figure S2: KEGG pathway enrichment analysis.
