# Supplementary figures and images for: Transcriptomic and Targeted Metabolomics Analysis of Detached Lycium ruthenicum Leaves Reveals Mechanisms of Anthocyanin Biosynthesis Induction through Light Quality and Sucrose Treatments
Source: Metabolites. 2023 Sep 11;13(9):1004. doi: 10.3390/metabo13091004 (PMC10535117; doi:10.3390/metabo13091004)

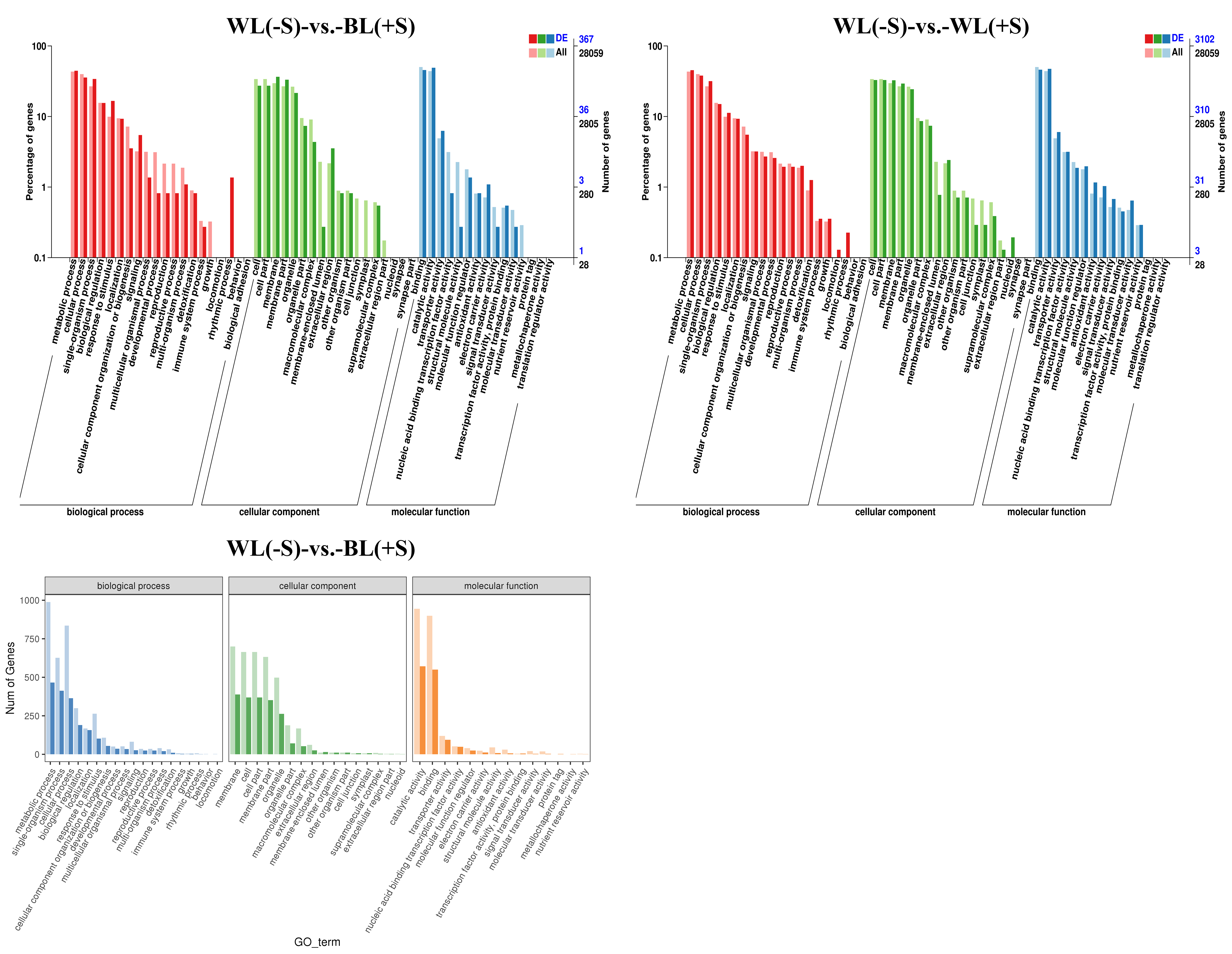

Supplement: Supplementary file 1 [file metabolites-13-01004-s001.zip › Figure S1.jpg]

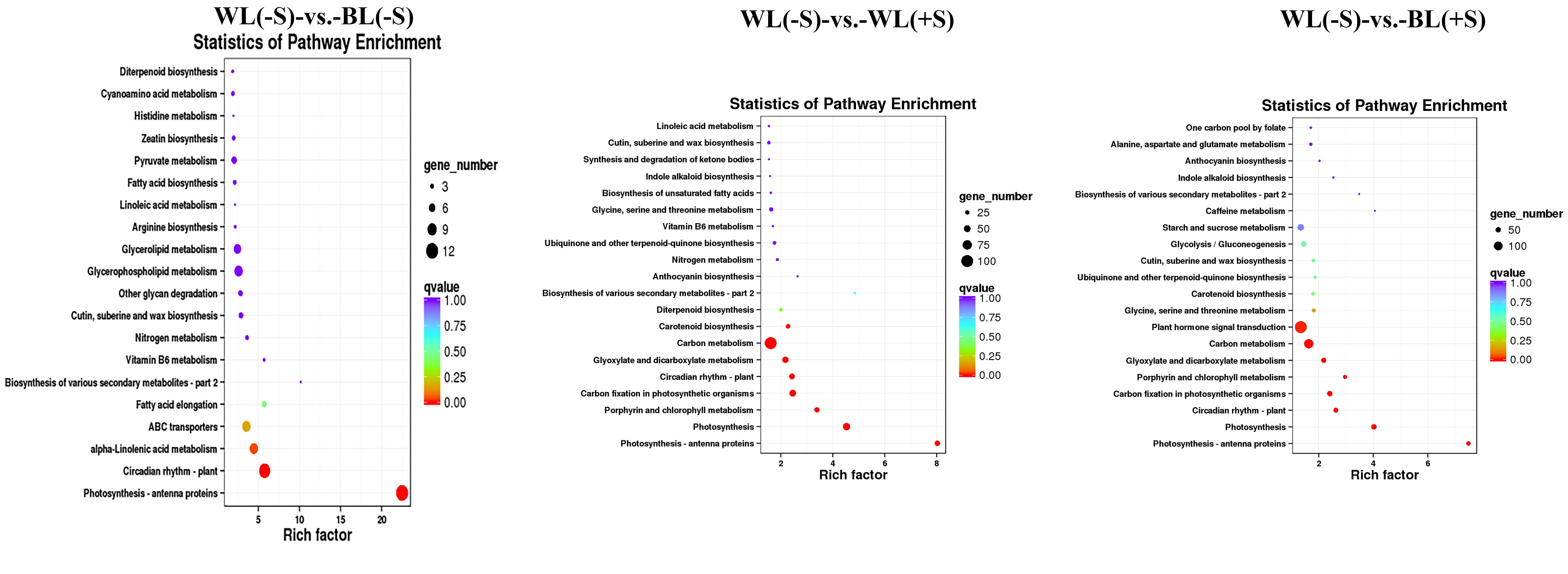

Supplement: Supplementary file 1 [file metabolites-13-01004-s001.zip › Figure S2.jpg]
